# Causal associations between chronic heart failure and the cerebral cortex: results from Mendelian randomization study and integrated bioinformatics analysis

**DOI:** 10.3389/fcvm.2024.1396311

**Published:** 2024-07-04

**Authors:** Liqi Peng, Huzhi Cai, Yanping Tang, Fang Zhou, Yuemei Liu, Zelin Xu, Qingyang Chen, Xinyu Chen

**Affiliations:** ^1^ The First Clinical College of Chinese Medicine, Hunan University of Chinese Medicine, Changsha, China; ^2^ International Medical Department, The First Affiliated Hospital of Hunan University of Chinese Medicine, Changsha, China; ^3^ College of Integrative Medicine, Hunan University of Chinese Medicine, Changsha, China; ^4^ Health Management Department, The First Affiliated Hospital of Hunan University of Chinese Medicine, Changsha, China; ^5^ Department of Cardiovascular Medicine, The First Affiliated Hospital of Hunan University of Chinese Medicine, Changsha, China; ^6^ Preventive Treatment Center, The First Affiliated Hospital of Hunan University of Chinese Medicine, Changsha, China; ^7^ Intensive Care Unit, The First Affiliated Hospital of Hunan University of Chinese Medicine, Changsha, China

**Keywords:** chronic heart failure, cerebral cortical structure, Mendelian randomization, causal association, integrated bioinformatics

## Abstract

**Background:**

Chronic heart failure (CHF) patients exhibit alterations in cerebral cortical structure and cognitive function. However, the mechanisms by which CHF affects cortical structure and functional regions remain unknown. This study aims to investigate potential causal relationship between CHF and cerebral cortical structure through Mendelian randomization (MR).

**Methods:**

The research utilized genome-wide association studies (GWAS) to explore the causal association between CHF and cerebral cortical structure. The results were primarily analyzed using the inverse-variance weighted (IVW). The reliability of the data was verified through horizontal pleiotropy and heterogeneity analysis by MR-Egger intercept test and Cochran's *Q*-test, respectively. Replication analysis was conducted in the Integrative Epidemiology Unit (IEU) OpenGWAS project for further validation. In addition, we collected mediator genes that mediate causality to reveal potential mechanisms. Integrated bioinformatics analysis was conducted using the Open Target Genetics platform, the STRING database, and Cytoscape software.

**Results:**

The IVW results did not reveal any significant causal association between genetically predicted CHF and the overall structure of the cerebral cortex or the surface area (SA) of the 34 functional regions of the cerebral cortex (*P* > 0.05). However, the results revealed that CHF increased the thickness (TH) of pars opercularis (IVW: *β *= 0.015, 95% CI: 0.005–0.025, *P* *= *3.16E-03). Replication analysis supported the causal association between CHF and pars opercularis TH (IVW: *β *= 0.02, 95% CI: 0.010–0.033, *P *= 1.84E-04). We examined the degree centrality values of the top 10 mediator genes, namely CDKN1A, CELSR2, NME5, SURF4, PSMA5, TSC1, RPL7A, SURF6, PRDX3, and FTO.

**Conclusion:**

Genetic evidence indicates a positive correlation between CHF and pars opercularis TH.

## 1 Introduction

Chronic heart failure (CHF) is the severe and final stage in the development of most cardiovascular diseases (CVDs), characterized by dyspnea, decreased exercise tolerance, and edema in patients. According to epidemiological surveys, there are currently over 64 million patients with CHF worldwide (1). Within 5 years, the readmission and mortality rates of patients can be as high as 80% and 50%, respectively (2). With the aging trend of society, the current prevalence of CHF among Chinese residents aged ≥35 years is 1.3%, and the number of patients is as high as 8.9 million (3, 4). By 2030, the prevalence rate in the elderly population is expected to reach 8.5% (5). Statistics indicate that CHF patients in China are hospitalized an average of 3.3 times per year, with a per capita cost of $4,982 for inpatient and outpatient treatment (6). Despite the progress that has been made in treating CHF, plenty of patients still experience unsatisfactory outcomes and poor prognosis.

The cerebral cortex is a layer of gray matter that covers the surface of the cerebral hemispheres and is composed primarily of neuronal cells (7). It is widely acknowledged that the structure of the cerebral cortex is responsible for a number of higher cognitive functions in humans, including the processes of thinking, learning, memory, and language (8, 9). The human cerebral cortex is characterized by two main parameters: surface area (SA) and thickness (TH), which are regulated by multiple genes (10). In-depth studies related to the cerebral cortex can enhance our comprehension of alterations in cortical structure during disease progression and throughout the lifespan. The “heart-brain axis” refers to the bidirectional communication network between the heart and the central nervous system (CNS), involving hemodynamic changes and neuronal signaling. This concept involves a tight heart-brain feedback interaction and is highly complex (11, 12). Researches have demonstrated that more than 50% of patients with CHF experience secondary brain damage, which can manifest as autonomic damage, cognitive dysfunction, and neuropsychological deficits (13). In addition to their relation to the grey matter structure of the subcortex and brainstem, these varying degrees of brain damage also involve the integrity of cerebral cortical structure (14). Several scientific studies have examined the structure of the cerebral cortex, and the cortical regions of patients with CHF have been roughly examined using magnetic resonance imaging (MRI) (15–17). However, the assessment of altered cerebral cortical structure in specific regions has not been effective. The causal associations between CHF and cerebral cortex remain unknown.

Mendelian randomization (MR) analysis, as a naturalistic randomized controlled trial, is an effective approach to evaluate potential causal associations between specific exposure factors and outcomes. In contrast to randomized controlled trials (RCTs), MR analysis employs single nucleotide polymorphism (SNP) as an instrumental variable (IV) based on Mendel's law of independent assortment. This approach can effectively aid in establishing a causal association between phenotype and disease (18, 19). Additionally, MR analysis can address the limitations of traditional epidemiological studies that are susceptible to potential confounding factors. As a result, MR study allows for obtaining more plausible causal associations and a stronger ability to argue for etiological inferences (20). Currently, the joint application of genome-wide association studies (GWAS) and biological big data has become a new trend in scientific research. This approach provides an opportunity to explore etiological associations from a genetic perspective and lays the foundation for the wide application of MR analysis (21). In recent years, researchers from both domestic and international settings have investigated the impact of diseases, circulating biomarkers, and behaviors on the structure of the cerebral cortex through the use of MR studies. These studies have confirmed that obesity (body mass index (BMI) and waist-to-hip ratio (WHR)), elevated blood lipid levels, and sleep disorders (insomnia and shorter sleep duration) are associated with alterations in the structure of the cerebral cortex (22–24). In this study, we employed publicly available GWAS summary statistics to preliminarily investigate the effects of CHF on the structure of the cerebral cortex and to explore its correlation with cerebral cortical SA and TH. Subgroup analyses were conducted according to different functional regions of the cerebral cortex, providing new insights into the effects of CHF on cerebral cortical structure and its underlying mechanisms.

## 2 Materials and methods

### 2.1 Study design

**Figure 1:**
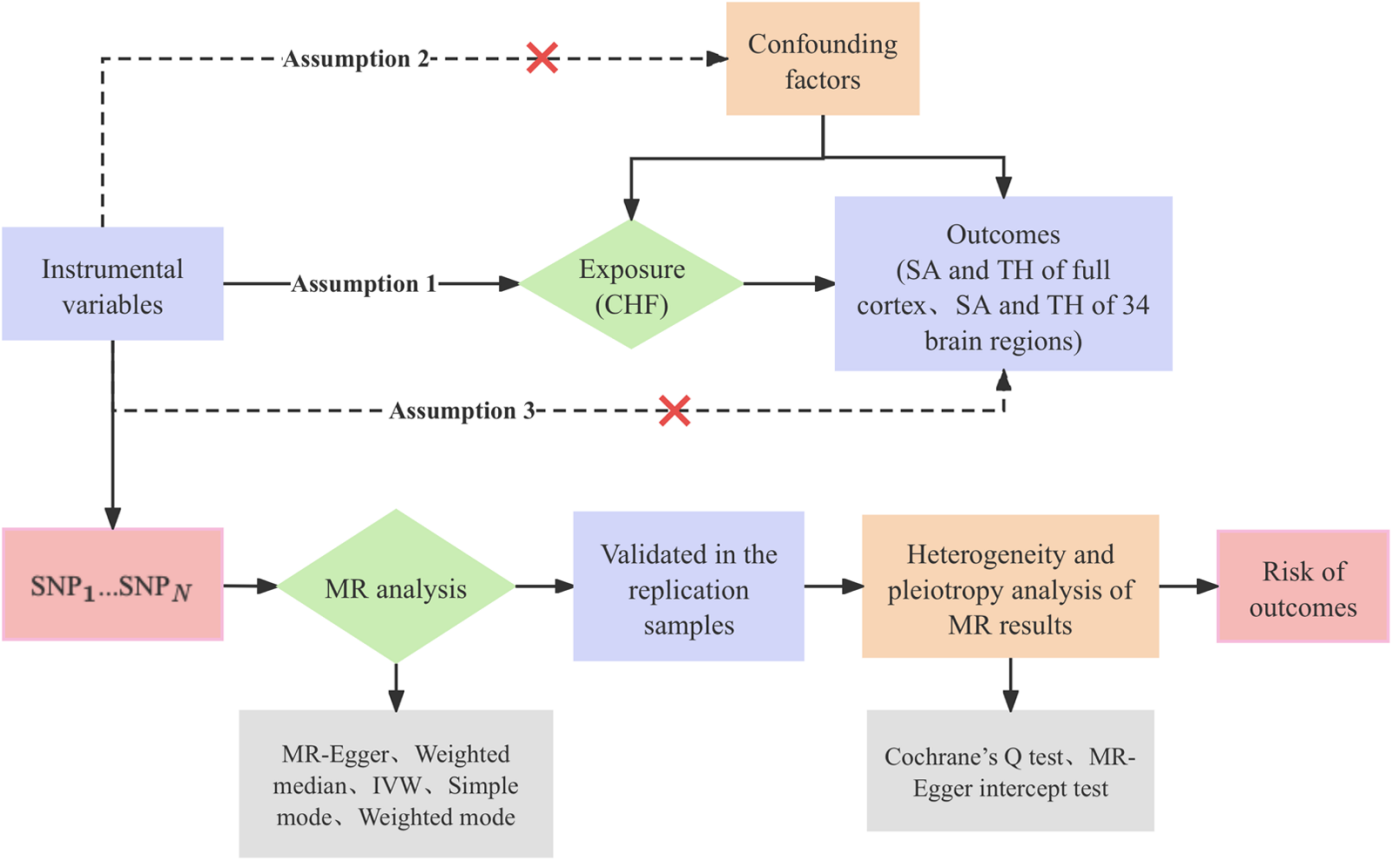
Research design flow chart. CHF, chronic heart failure; SA, surface area; TH, thickness; SNP, single nucleotide polymorphism; MR, Mendelian randomization; IVW, inverse-variance weighted.

This study was conducted in accordance with the established standards for reporting on two-sample MR analyses (25). The genetic IVs for phenotypes should meet the three crucial assumptions (26): (a) There is a robust and strong correlation between IVs and CHF. (b) IVs are not affected by confounding factors that may influence the relationship between CHF and cerebral cortical structure. (c) IVs affect cerebral cortical structure only through the onset of CHF, but not through other pathways. The study investigated CHF as the exposure factor and measured three outcomes in the following order: overall cortical structure, SA of functional regions of the cerebral cortex, and TH of functional regions of the cerebral cortex. We initially conducted MR studies with the objective of comprehensively analyzing the causal associations between CHF and the cerebral cortex. Subsequently, we proceeded to perform replication analysis with the intention of validating the identified causal association. Figure 1 shows the study design flowchart.

### 2.2 Data sources

#### 2.2.1 Exposure: CHF

The GWAS summary statistics for CHF were obtained from the Cardiovascular Disease Knowledge Portal (CVDKP) database, which included 26 cohort studies (27). The study consisted of 17 population-based cohorts that participated in the Heart Failure Molecular Epidemiology for Therapeutic Targets Consortium (HERMES), comprising 38,780 cases and 893,657 controls, as well as nine case-control studies with 8,529 cases and 36,357 controls. The study participants in this GWAS study were of European ancestry, totaling 47,309 cases and 93,014 healthy controls. In this meta-analysis study, Shah et al. employed logistic regression (LR) to analyze the association between autosomal SNPs and heart failure (HF). The overall effect model was evaluated using inverse-variance weighted (IVW). SNPs exhibiting linkage disequilibrium (LD) were excluded based on the principle of “*P*  <  5  ×  10^−8^, *r *^2 ^ <  0.1”. The researchers ultimately identified 12 independent genetic variants associated with HF risk by analyzing 11 genomic loci, providing new insights into the understanding of HF etiology.

#### 2.2.2 Outcome: cerebral cortex structure

The outcome dataset utilized in this study was derived from the GWAS summary statistics published in March 2020 by the Enhancing Neuro Imaging Genetics Through Meta-Analysis (ENIGMA) consortium (http://enigma.ini.usc.edu). The study performed a meta-analysis of MRI data from 51,665 individuals from 60 cohorts worldwide (28). The GWAS summary statistics included the total cerebral cortex SA and average TH, as well as 34 cortical regions with known function defined according to the Desikan-Killiany cortical atlas. The subject population was approximately 94% of European ancestry. Grasby et al. identified a total of 70 categories of cortical phenotypes and 306 statistically significant loci through GWAS analyses and multiple statistical comparisons (*P* < 5 × 10^−8^). After multiple statistical correction tests, the study confirmed that only 187 genomic loci were associated with cortical SA and 12 genomic loci were associated with cortical TH. The proportion of SA phenotypic variation that could be explained by common genetic variants was greater than that of TH phenotypic variation, both in terms of the overall structure of the cerebral cortex and in terms of the 34 specific functional regions.

#### 2.2.3 CHF replication samples

The CHF replication samples were derived from GWAS summary statistics (ebi-a-GCST90018806) included in September 2021 by the Integrative Epidemiology Unit (IEU) OpenGWAS project (https://gwas.mrcieu.ac.uk) (29). A total of 14,262 cases and 47,189 controls were included in the study, with data from BioBank Japan (BBJ), the UK Biobank (UKB) and FinnGen. The CHF replication samples comprised 10,540 cases and 168,186 controls derived from BioBank Japan, and 6,526 cases and 350,289 controls derived from the UK Biobank and FinnGen databases. The subject population consisted of individuals of Japanese and European ancestry. In this study, Sakaue et al. conducted a genome-wide association study of 220 human phenotypes from BioBank Japan, successfully replicating 94.2% of the variant loci in the European population. A cross-population meta-analysis was conducted with 196 human phenotypes from the UK Biobank and 128 human phenotypes from FinnGen. The meta-analysis identified 1,730 disease-associated loci, 12,066 biomarker-associated loci, and 1,018 drug-associated loci, respectively, which extended the genetic association map in non-European populations.

### 2.3 Selection of instrumental variables

Instrumental variables significantly associated with CHF were screened from the GWAS summary statistics with a threshold of “*P* < 5 × 10^−8^”. SNPs that were not affected by linkage disequilibrium and were independent of each other were screened using “*r*² < 0.001 and Clump distance >10,000 kb” as thresholds (30). Additionally, the formula $F\;=\;R^{2}\left(n-2\right)/\left(1-R^{2}\right)$ was employed to calculate the *F* statistic in order to detect any potential bias due to weak IVs. Only IVs with *F* statistics greater than 10 were included in this study. In accordance with the exclusivity assumption of MR analysis, SNPs associated with cerebral cortical structure should be excluded (*P* < 5 × 10^−8^). Regarding the independence assumption, secondary phenotypes were identified for each SNP using the PhenoScanner V2 website (http://www.phenoscanner.medschl.cam.ac.uk/) (31), and SNPs associated with confounding factors (type 2 diabetes, body mass index, blood pressure, and coronary heart disease) (32) were excluded.

### 2.4 Functional exploration of mediator genes

In order to identify the core mediator genes regulating the causal association between CHF and cerebral cortex structure, and to reveal the potential biological pathways between CHF and brain dysfunction, we conducted an integrated bioinformatics analysis. Genes that are functionally associated with genetic variation are referred to as mediator genes. The Open Target Genetics platform (33) is a free and open-source tool that highlights statistical evidence centered on genetic variation. It utilizes human genetics and genomics data for systematic drug target identification and prioritization, exploring the intrinsic associations between traits, variants, and genes, and identifying potential drug targets (34). The Open Target Genetics platform (https://genetics.opentargets.org/) was employed to genetically annotate the genetic variants utilized as IVs, with the subsequent construction of a protein-protein interaction (PPI) network based on these mediator genes in the STRING database (version 12.0) (https://cn.string-db.org/). The Cytoscape software (version 3.9.1) was used to visualize and analyze the complex network based on the node degree and integrated score of target proteins in the PPI network. The topology of the network graph was analyzed using the CytoHubba plug-in. Each mediator gene was assigned a value by the topological network algorithm, and the hub genes and the subnetwork were identified after sorting. We conducted Gene Ontology (GO) terms and Kyoto Encyclopedia of Genes and Genomes (KEGG) pathway enrichment analysis using the Bioinformatics Online Cloud Platform (https://www.bioinformatics.com.cn/) in order to present the results of the functional annotation of mediator genes in a more intuitive manner.

### 2.5 Statistical analysis

R studio software (version 4.3.2) and the TwoSampleMR package (version 0.5.6) were used for statistical analysis. We utilized selected SNPs as IVs to evaluate the causal relationship between CHF and cerebral cortex structure. Five methods were employed: MR-Egger, inverse-variance weighted, weighted median, simple mode, and weighted mode. The results of the MR study were mainly dominated by the IVW analysis, while the MR-Egger method and the weighted median approach were able to enhance the estimation accuracy of the IVW analysis. The cluster-heatmap format of IVW analysis results was generated by the Bioinformatics Online Cloud Platform. The replication MR analysis procedure was identical to that employed in the discovery stage.

Instrumental variables from different analytical platforms, experiments, populations, etc., may be heterogeneous and thus affect the results of MR analyses. In addition, if IVs influence the occurrence of an outcome through factors other than the exposure factor, this indicates that the IVs are pleiotropic. Pleiotropy can lead to a violation of the assumptions of independence and exclusivity. Therefore, if there is a causal relationship between CHF and cerebral cortex structure, it is necessary to confirm the reliability of the findings through the test of heterogeneity and horizontal pleiotropy. In this study, Cochran's *Q*-test was used to test for heterogeneity among the included studies (*P*-value < 0.05 indicates heterogeneity). The MR-Egger regression test was used to determine the presence of horizontal pleiotropy (*P*-value < 0.05 indicates horizontal pleiotropy). A leave-one-out (LOO) analysis was employed in the sensitivity analysis to evaluate whether a single SNP had an effect on the association between CHF and cerebral cortex structure. The leave-one-out method entailed the exclusion of a single SNP one at a time, with the remaining SNPs subsequently subjected to an aggregate effect calculation.

Furthermore, the Bonferroni correction was employed to account for the multiplicity of test results. For each multiple test, the new significance level was set at *P* < 0.05/*n*, where *n* represents the number of results (35). Consequently, a *P*-value of less than 2.5 × 10^−2^ (0.05/2) was deemed statistically significant in the estimation of the overall structure of the cerebral cortex, whereas a *P*-value of less than 7.35 × 10^−4^ (0.05/68) was considered statistically significant in the estimation of the 34 specific functional regions of the SA and TH. A *P*-value of less than 0.05 was deemed suggestive of an association and a *P*-value of less than 0.01 was considered suggestive of a stronger association.

## 3 Results

### 3.1 Details of instrumental variables

This study screened for SNPs that differed significantly on a genome-wide basis using the completed GWAS database for CHF. A total of nine SNPs were finally included (Supplementary Table S1). The median value of the *F*-statistics for the IVs was 34.87, with a range of 30.89–83.10. It is noteworthy that all *F*-statistics were greater than 10. This suggests a significant correlation between the IVs and the exposed factors represented by CHF. The results of the MR analyses are not significantly affected by the inclusion of weak IVs.

### 3.2 Results of MR analysis

**Figure 2:**
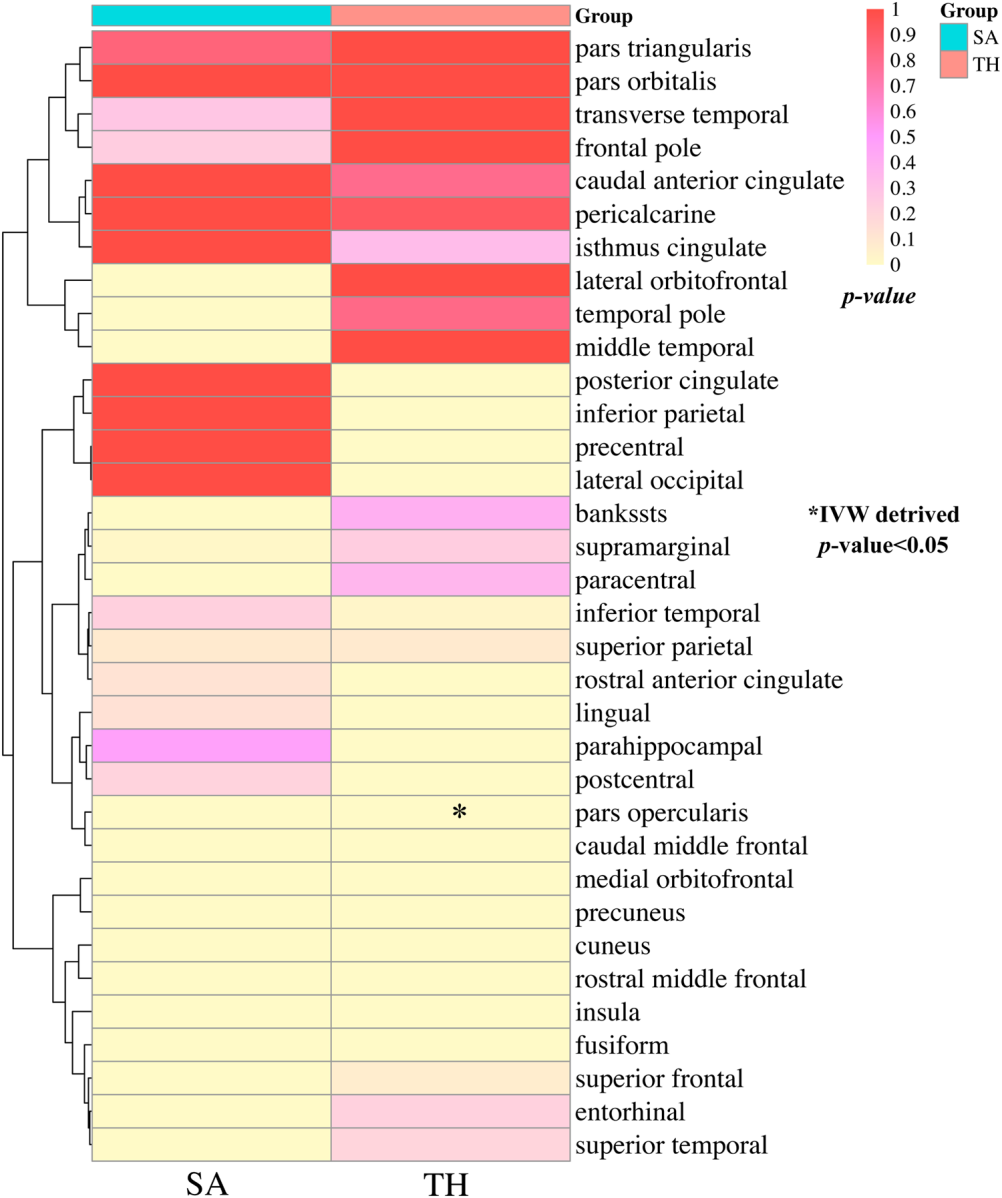
Clustering heat maps of IVW results of causality between CHF and SA and TH of cerebral cortex. SA, surface area; TH, thickness; IVW, inverse-variance weighted.

**Figure 3:**
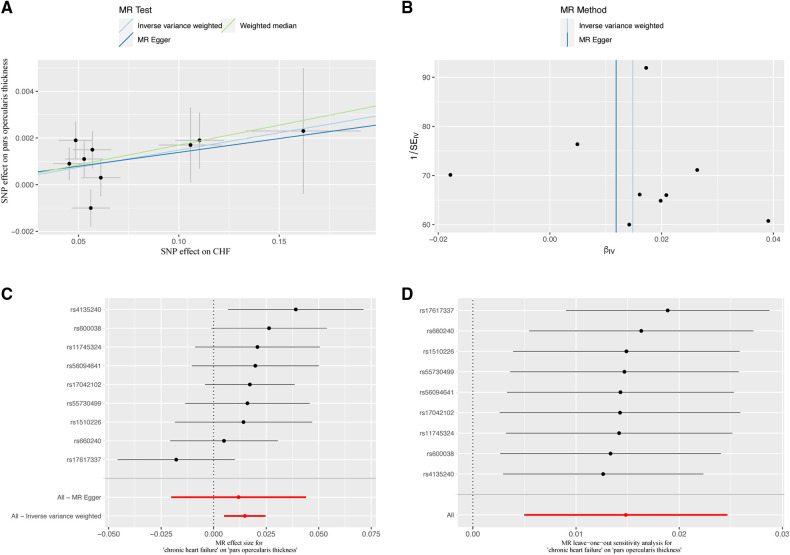
Causal relationship of CHF in predicting pars opercularis TH. (**A**) Scatter plot; (**B**) funnel diagram; (**C**) individual SNP causal effect diagram; (**D**) leave-one-out sensitivity analysis. MR, Mendelian randomization; SNP, single nucleotide polymorphism; CHF, chronic heart failure.

**Figure 4:**
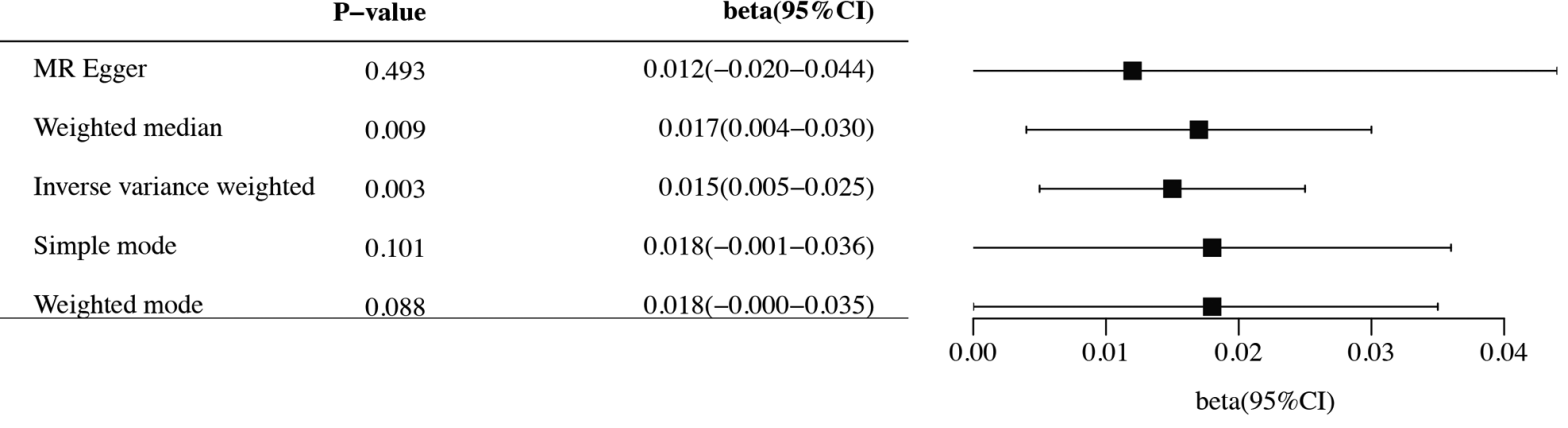
Forest diagram of causal association between CHF and pars opercularis TH. MR, Mendelian randomization; CI, confidence interval.

The study conducted comprehensive MR analyses to assess the causal associations between genetically predicted CHF and the overall structure of the cerebral cortex, SA and TH of the 34 functional regions of the cerebral cortex. The results showed no statistically significant association between CHF and the overall structure of the cerebral cortex (IVW: *β*_SA_ = 247.489, 95% CI_SA_: −1,799.191–2,294.168, *P*_SA _= 0.813; IVW: *β*_TH_ = −0.009, 95% CI_TH_: −0.019–0.001, *P*_TH _= 0.066) (Supplementary Table S2). Figure 2 presents the changes in IVW analysis results between CHF and the SA/TH of the 34 functional regions of the cerebral cortex using a cluster-heatmap format. The IVW analysis did not find a significant causal relationship between genetically predicted CHF and the SA of the 34 functional regions (*P* > 0.05) (Supplementary Table S3). Nevertheless, in specific functional region analyses, a potential causal relationship between CHF and pars opercularis TH was identified (IVW: *β *= 0.015, 95% CI: 0.005–0.025, *P* *=* 3.16E-03) (Supplementary Table S4). The IVW results indicated a positive correlation between CHF and alterations of pars opercularis TH. Specifically, the results of the weighted median method and the IVW analysis were similar and both were statistically significant (weighted median: *β* = 0.017, 95% CI: 0.004–0.030, *P* *=* 9.50E-03). Figures 3A,B illustrate the scatter plot and the funnel plot of the causal relationship between CHF and pars opercularis TH, respectively. The asymmetry in the funnel plot may be due to factors such as clinical or methodological heterogeneity between studies that do not introduce a significant degree of bias into the results. The results of the meta-analysis of the causal relationship between CHF and pars opercularis TH are presented in Figure 4.

The causal relationship between CHF and pars opercularis TH was successfully replicated in the MR analysis of the IEU OpenGWAS project. The results of the IVW analysis demonstrated a positive correlation between CHF and pars opercularis TH (*β* = 0.02, 95% CI: 0.010–0.033, *P* = 1.84E-04) (Supplementary Table S5). The replication analysis yielded results consistent with those of previous MR studies, thereby providing support for the reliability of the present findings.

### 3.3 Sensitivity analysis

We assessed the magnitude of heterogeneity and horizontal pleiotropy by using Cochran's *Q*-test and MR-Egger intercept test. The Cochran's *Q*-test statistic was not statistically significant (*P* > 0.05), indicating no heterogeneity among the SNPs associated with CHF (Supplementary Tables S6–S8). The MR-Egger intercept results indicated no horizontal pleiotropy between CHF and pars opercularis TH (*P* > 0.05) (Supplementary Tables S9–S11), providing strong evidence for a direct causal relationship. The leave-one-out analysis demonstrated that no SNP could potentially drive the null causal effect of CHF on pars opercularis TH (Figures 3C,D).

### 3.4 Results of integrated bioinformatics analysis

**Figure 5:**
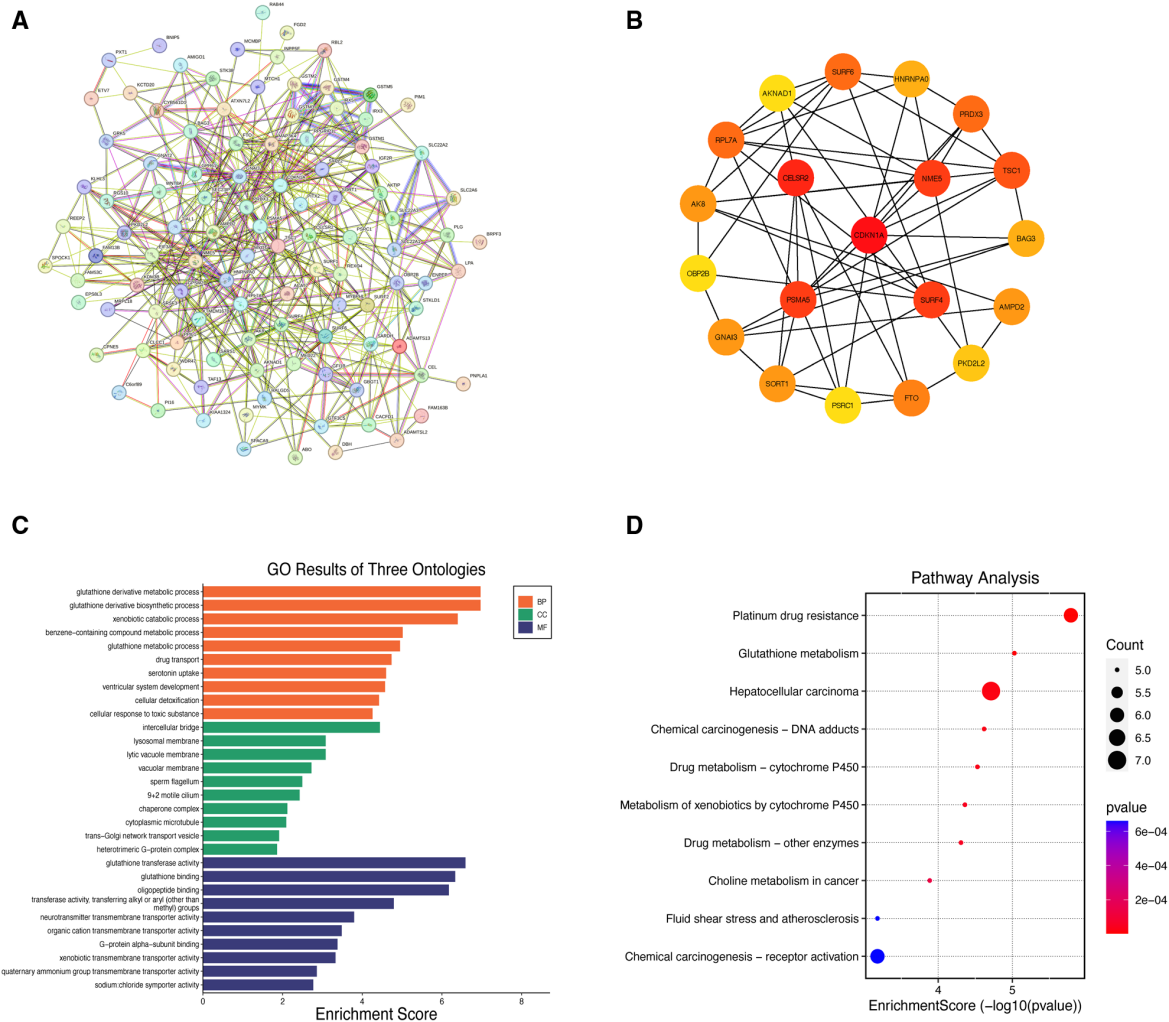
Results of integrated bioinformatics analysis. (**A**) Protein-protein interaction network of mediator genes; (**B**) diagram of the top 20 mediator genes interactions; (**C**) histogram of GO biological function analysis; (**D**) bubble diagram for KEGG pathway enrichment analysis. GO, gene ontology; KEGG, Kyoto encyclopedia of genes and genomes; BP, biological process; CC, cellular component; MF, molecular function.

This study identified the dominant instrumental loci for the causal associations between CHF and cerebral cortex structure and analyzed their genetic profiles. After eliminating duplicated sequences, we matched 9 independent SNPs with genetic variations of the mediator genes, and identified 107 mediator genes. The correspondence between SNPs and mediator genes is shown in Supplementary Table S12. The PPI network was constructed using the STRING database with a screening criterion of “Minimum Required Interaction Score >0.150” (Figure 5A). The network illustrates the correlation between mutation-associated mediator genes (Supplementary Table S13). The PPI network consisted of 107 nodes and 483 edges, with an average node degree of 9.03, an average local clustering coefficient of 0.427, and an enriched *P*-value < 1.0E-16. The PPI network data were detailed in Supplementary Table S14. The top 10 mediator genes with the highest degree centrality values were screened, which included CDKN1A, CELSR2, NME5, SURF4, PSMA5, TSC1, RPL7A, SURF6, PRDX3, and FTO (Figure 5B).

A total of 210 entries were obtained from GO terms analysis under the screening condition of “*P* < 0.05”, including 149 Biological Process (BP), 16 Cellular Component (CC), and 45 Molecular Function (MF) entries (Supplementary Table S15). Furthermore, 16 entries were obtained from KEGG pathway enrichment analysis (Supplementary Table S16). Figures 5C,D show the results of the GO terms histogram and the KEGG pathway enrichment analysis bubble plot. The mediator genes in the BP analysis were primarily concentrated in “glutathione derivative metabolic process”, “glutathione derivative biosynthetic process” and “xenobiotic catabolic process”. The CC analysis revealed that mediator genes were mainly enriched in “intercellular bridge”, “lysosomal membrane” and “lytic vacuole membrane”. Regarding MF analysis, the mediator genes were principally involved in “glutathione transferase activity”, “glutathione binding” and “oligopeptide binding”. The KEGG pathway enrichment analysis indicated that the mediator genes were primarily enriched in pathways related to “platinum drug resistance”, “glutathione metabolism” and “epatocellular carcinoma”.

## 4 Discussion

Patients with CHF frequently exhibit concomitant brain damage, which typically involves the integrity of cortical structure. In this study, a causal relationship between CHF and pars opercularis TH was determined through the use of two-sample MR analysis. The results of this study indicate that patients with CHF exhibit increased pars opercularis TH. The integrated bioinformatics analysis revealed that CHF may affect pars opercularis TH through 10 key mediator genes. Further KEGG enrichment analysis indicates that glutathione (GSH) metabolism may be a potential biological pathway by which CHF affects the structure of the cerebral cortex. This establishes a foundation for further elucidation of the relationship between CHF and neuropsychiatric disorders, including cognitive dysfunction, dementia, and depression.

The pars opercularis is a key component of the motor-linguistic area, situated posterior to the inferior frontal gyrus of the frontal lobe, as designated by Brodmann as area 44 (BA44) (36). The pars opercularis is essential for lexical extraction, language synthesis, memory, coordination of the oral muscles, and various cognitive functions (37–39). The normal function of the pars opercularis depends on the integrity of the cortex, while its volume is determined by both total SA and average TH. A review of the literature indicates that several factors regulate the SA and TH of the human cerebral cortex. Based on the radial unit hypothesis (40), cortical SA is determined by the number of neural progenitor cell proliferation units, while cortical TH is dependent on the number of cell divisions within each proliferation unit. In functional regions of the cerebral cortex, changes in cortical TH are primarily influenced by genetic factors and tend to decrease with age (41–43). Most neurodegenerative diseases (NDDs) and cerebrovascular diseases (CVDs) are characterized by cortical TH thinning (44). The study findings indicate that there is no significant causal relationship between CHF and the overall structure of the cerebral cortex. This may be attributed to the fact that cerebral glucose metabolism in patients with early CHF maintains a relative balance of compensatory increases and decreases, thus preventing a significant alteration in the overall structure of the cerebral cortex (45). In recent years, researchers have devoted considerable attention to the potential causal association between HF and the structure of functional cortical regions of the brain (46, 47). These findings have confirmed that frontal brain activity is reduced in HF, and that there is a significant positive correlation between left ventricular ejection fraction (LVEF) and overall density of the frontal cortex (48, 49). Notably, the conclusions drawn from subgroup analyses were opposite to the results of previous findings. Kumar et al. conducted a study that revealed a decrease in regional cortical TH in areas regulating automatic memory, cognitive, emotional, linguistic, and visual functions in patients with HF (50). Nevertheless, the present study found that CHF is associated with an increase in pars opercularis TH. This indicates that neuropsychiatric disorders, such as cognitive dysfunction, dementia, or depression, may not be the primary cause of thickening in the pars opercularis in individuals with CHF. Most patients with CHF often experience low cardiac output and sleep apnea, which can cause hypoxia/ischemia-induced brain damage. This damage can affect the TH of cortical structural regions of the brain (51). Baril et al. discovered obstructive sleep apnea generally alters gray matter structure and that high levels of hypoxemia are associated with increased TH of the left prefrontal cortex (52). Therefore, we propose the hypothesis that CHF may lead to hypoxemia, resulting in neuronal cell dysfunction, increased capillary permeability, accelerated division of neural progenitor cells, and ultimately compensatory hypertrophy of cerebral cortical structure. In addition, prolonged hypoxia in the brain stimulates the secretion of adrenaline (A) and norepinephrine (NE), leading to vasoconstriction. This exacerbates cerebral hypoxia, resulting in cerebral oedema and abnormal thickening of the cerebral cortex. In summary, further in-depth exploration is required to investigate the potential molecular mechanisms behind the complex changes that may occur in pars opercularis TH or other specific functional regions in the CHF population.

Integrated bioinformatics analysis identified 10 key genes, namely CDKN1A, CELSR2, NME5, SURF4, PSMA5, TSC1, RPL7A, SURF6, PRDX3, and FTO, which could help elucidate the potential mechanisms underlying the association between CHF and structural alterations in the cerebral cortex. The KEGG pathway enrichment analysis revealed that five mediator genes, which were closely related to the genetic variations, were enriched in the “glutathione metabolism” signaling pathway (*P* = 9.36E-06). GSH is a biologically active polypeptide composed of glutamic acid, cysteine, and glycine, containing a *γ*-amide bond and sulfhydryl groups (53). GSH is widely distributed in animals and plants and plays a crucial role in various biological processes, including DeoxyriboNucleic Acid (DNA) and protein synthesis, amino acid transport, gene expression, and cell proliferation and apoptosis (54, 55). In the context of CVDs, GSH serves as a marker for prophylactic antioxidant therapy and the risk of adverse cardiovascular events (56). Researches have demonstrated that GSH can not only scavenge reactive oxygen species via the glutathione peroxidase (GSH-Px) and glutathione S-transferase (GST) pathways, but also bind to free radicals to exert detoxifying and antioxidant effects, thereby attenuating myocardial cells apoptosis (57, 58). Several CVDs, including myocardial ischemia/reperfusion injury (MI/RI), coronary atherosclerotic heart disease (CHD), hypertension (HT), and myocardial infarction (MI), have been found to have a negative correlation with GSH levels (59–61). Meanwhile, GSH plays a vital role in the antioxidant defense system and in maintaining redox homeostasis in neuronal cells. A reduction in the concentration of GSH in neuronal cells has been demonstrated to be a significant contributing factor in the development of Alzheimer's disease (AD), Huntington's disease (HD), and Parkinson's disease (PD) (62–64). Based on this hypothesis, if GSH metabolism is abnormal and GSH levels are reduced, it may affect both the cardiovascular and central nervous systems, increasing the susceptibility of myocardial cells and neuronal cells to oxidative stress. This can trigger cellular degeneration, necrosis, and apoptosis, ultimately leading to alterations in cortical structure.

To the best of our knowledge, a similar MR study has been conducted previously. The results of the study by Hu et al. demonstrated a causal effect of HF on the SA of the caudal middle frontal lobule, insula lobule, precuneus lobule and superior parietal lobule (65). In contrast to the present study, the investigators employed a screening criterion of “*P* < 5 × 10^−6^” to identify SNPs that predicted HF as the final IVs included in the study. The discrepancy in screening criteria may have contributed to the disparate outcomes observed in the two studies. In addition to employing MR analysis, our study also investigated the potential mechanism of CHF on the structure of the cerebral cortex through integrated bioinformatics techniques. The utilization of MR analysis in this study enabled the avoidance of residual confounding factors and reverse causality, thereby compensating for the inherent limitations of observational studies. Genetic variants were identified at the time of conception and followed the principle of random assignment. In this study, genetic variants associated with CHF were employed as the exposure and cerebral cortical structure as the outcome in order to ascertain whether there was a causal effect between CHF and cerebral cortex structure. This approach effectively avoided reverse causality in this MR study. Besides, the replication analysis was conducted within the IEU OpenGWAS project with the objective of validating the MR results.

Inevitably, the current study still has certain limitations. The GWAS sample data utilized in this study were predominantly derived from populations of European ancestry. Therefore, the results cannot be used to establish causal associations between CHF and cerebral cortex structure in ethnically diverse populations. Moreover, the statistics on exposure factors selected during the study were pooled and did not categorize specific forms of CHF. The causal associations between cerebral cortical structure and CHF type and grade remains unclear. What's more, the present study did not address the severity of altered SA or TH of functional regions of the cerebral cortex. The present study employed integrated bioinformatics analysis to clarify the critical targets and potential molecular mechanisms involved in regulating the TH alteration of pars opercularis. Nevertheless, further experimental studies are required to substantiate these findings.

## 5 Conclusion

In summary, this study provides preliminary genetic evidence supporting a positive correlation between CHF and pars opercularis TH through MR analysis. This study contributes to the in-depth examination of the “heart-brain axis” theory. Further investigation is necessary to comprehend the association and potential mechanism of action between CHF, cerebral cortical structure, and altered brain function.

## Data Availability

The original contributions presented in the study are included in the article/**Supplementary Material**, further inquiries can be directed to the corresponding author.
